# Toxicity Assessment of Organophosphate Flame Retardants Using New Approach Methodologies

**DOI:** 10.3390/toxics13040297

**Published:** 2025-04-11

**Authors:** Maryam Pyambri, Joaquim Jaumot, Carmen Bedia

**Affiliations:** 1 Environmental Chemistry Department, Institute of Environmental Assessment and Water Research (IDAEA-CSIC), Jordi Girona 18-26, 08034 Barcelona, Spain; maryampramani@gmail.com (M.P.); joaquim.jaumot@idaea.csic.es (J.J.); 2 Department of Analytical Chemistry, Faculty of Chemistry, University of Barcelona, Martí i Franquès 1-11, 08028 Barcelona, Spain

**Keywords:** organophosphate flame retardants, New Approach Methodologies, in vitro methods, cell cultures, omics, toxicity

## Abstract

Organophosphate flame retardants (OPFRs) have increasingly replaced polybrominated diphenyl ethers (PBDEs) in various consumer products and raw materials, due to regulatory restrictions on PBDEs. However, concerns about the toxicity and environmental persistence of OPFRs are growing. This review summarizes current research on the toxicity of OPFRs, with a focus on New Approach Methodologies (NAMs) that aim to eliminate the need for animal testing. NAMs, including in vitro models, omics technologies, and computational methods, provide valuable insights into the cellular and molecular effects of OPFR exposure. Evidence suggests that OPFRs may disrupt multiple organ systems, including the nervous, hepatic, pulmonary, reproductive, and endocrine systems. Additionally, the metabolic transformation of OPFRs can increase their toxicity, raising concerns about long-term exposure risks. While NAM studies provide valuable insights, further research is needed to refine risk-assessment frameworks and improve our understanding of the long-term effects of OPFR exposure, particularly at concentrations found in the environment. This new knowledge will help develop more accurate regulatory guidelines and ensure the better protection of public and environmental health.

## 1. Introduction

Flame retardant chemicals are frequently incorporated into consumer products and raw materials to slow down combustion and comply with fire safety regulations. However, since many of these chemicals are simply mixed in rather than chemically bonded to the material, they can gradually escape into the environment [1,2].

In the 1970s, polybrominated diphenyl ethers (PBDEs) were widely used in furniture, children’s products, and electronics. Studies revealed their persistence, toxicity, and potential to accumulate in living organisms, leading to bans in 2004 by the European Commission and California [3]. Following these actions, the United States EPA agreed in the same year to phase out pentabromodiphenyl ether (PentaBDE) and octabromodiphenyl ether (OctaBDE), later including decabromodiphenyl ether (DecaBDE) in 2009. Reducing PBDEs due to health concerns led to the increased use of organophosphate flame retardants (OPFRs) as substitutes, potentially resulting in greater human exposure [4]. Currently, OPFRs are among the most common flame retardants, frequently found in furniture, textiles, mattresses, and electronics [5]. In terms of the chemical structure, OPFRs are categorized into three main types: chlorinated OPFRs, alkyl-OPFRs, and aryl-OPFRs. Aryl and alkyl-OPFRs, which have higher molecular masses, tend to be more hydrophobic and strongly bind to sediment and soil [6,7]. In contrast, chlorinated OPFRs are more water-soluble, posing a threat to aquatic animals, including both animals and plants, by spreading in ecosystems and affecting their growth and physiological processes [6,8]. These chemicals are physically incorporated into the end products, which results in release during use due to volatilization, abrasion, and leaching [9].

Rainfall, industrial discharges from factories, and wastewater treatment plants (WWTPs), are the main ways that OPFRs enter aquatic and terrestrial ecosystems [7,10,11]. Due to this, these chemicals are widespread and often found in dust, indoor air, the atmosphere, surface water, sediment, and soil [12,13,14,15,16].

For wildlife exposed to OPFRs, ingestion is the primary absorption route. OPFRs can be absorbed by terrestrial birds and mammals through food and water [17]. In aquatic animals, OPFRs from the surrounding water mostly enter the body through the gills and skin, rather than via the food chain [9,16,18].

In humans, inhalation, ingestion, and dermal contact are the main exposure pathways [2]. Most assessments of human exposure to OPFRs via inhalation rely on the concentrations of OPFRs detected in indoor environments, such as homes, offices, and public buildings, such as libraries and schools [12,17]. These studies evaluate health risks by investigating OPFRs in indoor dust and air, where long-term exposure may affect human health [19,20,21]. To enhance clarity, Table 1 provides the full chemical names of the OPFRs mentioned in this review, along with relevant references. In these matrices, the most frequently identified OPFR compounds in indoor air and dust are TPhP, TBP, TBOEP, TCEP, and TCIPP [13,19,22]. OPFRs are present in the atmosphere. However, their effects on respiratory function are underestimated due to their low concentrations (1370 to 20,300 pg/m^3^) [7]. Additionally, OPFRs are also found in drinking water and food packaging, making ingestion a notable way people might be exposed [23,24,25,26]. Moreover, OPFRs can enter the body through skin contact with contaminated water or products [27].

Under REACH (Registration, Evaluation, Authorisation, and Restriction of Chemicals), the European Union restricts some OPFRs to protect human health and the environment. Among these are TCEP, TDCPP, and TCPP, which have raised concerns due to their persistence, bioaccumulation, and potential health risks.

**Figure 1:**
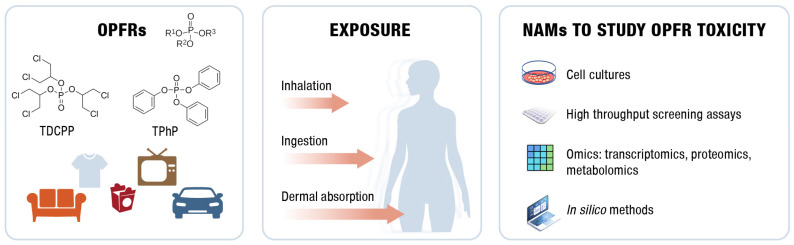
Diagram illustrating the occurrence and exposure pathways of OPFRs, along with the main NAMs used to assess their toxicity.

Studying the toxic effects of OPFRs is essential for understanding their cellular mechanisms of action and evaluating potential risks to human health. While some studies have relied on animal models, which offer valuable insights into OPFR toxicity, in vivo experimentation is costly, labor-intensive, time-consuming, and subject to ethical concerns. To address these challenges, New Approach Methodologies (NAMs) are gaining momentum in environmental research as an alternative to animal testing. This approach aligns with the principles of the REACH regulation, which encourages reducing animal testing, and the EU Directive 2010/63/EU, which promotes alternative methods for scientific research [63]. These methods include cell cultures, omics technologies, and in silico modeling, aimed at providing accurate and cost-effective results while avoiding the ethical issues associated with traditional animal-based research [64]. These studies help researchers understand mechanisms of action, including disruptions in gene expression, cell signaling, alterations in metabolism, and interactions with cellular receptors. They also allow for the detection of phenotypic features, such as membrane alterations, cytokine release, oxidative stress, DNA damage, and various forms of cell death, among other valuable insights. Meanwhile, omics technologies provide insights into molecular-level changes, including shifts in gene and protein expression, while computational models predict molecular interactions. Together, these NAM methods offer comprehensive insights into biological effects without the need for animal testing [65]. Figure 1 illustrates the occurrence and exposure pathways of OPFRs, along with the main NAMs used to evaluate their toxicity.

This review aims to summarize scientific studies investigating the toxicological effects of OPFRs employing NAMs. First, the review will explore the common methodologies used in these studies, followed by an analysis of the research results organized by the studied organs. Finally, the review will discuss the key conclusions and future perspectives in OPFR toxicology research.

## 2. New Approach Methodologies to Study OPFR Toxicity

This section outlines the most commonly used NAMs for studying the effects of OPFRs, focusing on the biological models and the toxicological methods employed. Supplementary Table S1 provides detailed information on the studies reviewed, including the biological models, OPFRs and doses used, as well as the main findings.

### 2.1. Biological Models

The primary biological models used as NAMs are cell cultures, which serve as essential in vitro systems for studying cellular responses, disease mechanisms, and toxicological effects under controlled conditions [66]. By selecting a specific cell line, researchers can model organ-specific effects, providing insight into potential impacts in a living organism. Most of the research on OPFRs has been conducted using traditional cell cultures involving growing cells adhered to a plastic surface in culture flasks. Both cancer cell lines and primary cells have been used to better replicate the functions of the studied organs. Cancer cell lines typically grow rapidly and are easy to culture, making them advantageous for toxicology studies. For instance, cancer cell lines, such as SH-SY5Y (human neuroblastoma) [32], MCF7 (human breast adenocarcinoma) [32], A549 (human lung carcinoma) [40], JEG-3 (placental choriocarcinoma) [35], and HepG2 (human hepatocellular carcinoma) [37], have been used as models to investigate the impact and mechanisms of OPFRs in different organs. However, results should be interpreted with caution, as they reflect cancer cell behavior rather than normal physiology. In contrast, primary cells are more challenging to culture but provide a more physiologically relevant model, as they are usually derived from healthy human donors or animal models. Primary cultures, including cortical neurons [29], microglia [65], hepatocytes [51], and embryonic-stem-cell-derived models from both humans and mice [29], have been employed to study OPFR effects in non-cancerous tissues, providing a more physiologically relevant perspective.

Traditional monolayer cell cultures differ significantly from the in vivo environment. To enhance physiological relevance, three-dimensional (3D) culture systems—such as spheroids and organoids—offer more realistic models that better mimic in vivo conditions [67]. However, few studies on OPFR toxicity have utilized 3D cultures. Notably, Negi et al. employed a 3D hepatospheroid model grown in agarose to investigate the effects of EHDPP [37], and Hogberg et al. investigated the neurotoxicity of four OPFRs using 3D brain neurospheres [42].

Although the strict definition of NAMs focuses on non-animal models, it is worth noting that some studies on OPFR toxicity have employed whole-organism models, such as zebrafish (*Danio rerio*) embryos (up to 5 days post-fertilization) or nematodes (e.g., *Caenorhabditis elegans*), as alternatives to traditional vertebrate animal testing. Since these organisms are not classified as protected animals under many regulatory frameworks, they offer a more ethically acceptable approach. These models allow for rapid and cost-effective screening compared to conventional rodent models and provide valuable insights into developmental effects and molecular pathways. Regarding OPFR studies, research on zebrafish embryos has primarily focused on endocrine disruption and developmental toxicity [68], while research using *C. elegans* has focused on neurological, growth, and reproductive toxicity [69].

### 2.2. Toxicity Assessment Methods

A range of in vitro assays has been employed to assess the cytotoxic effects of OPFRs. Some of these assays can be adapted to a multi-well plate format, which enables the high-throughput screening of these compounds at many concentrations using multiple replicates. Most studies have employed cell viability tests, including the 3-(4,5-dimethylthiazol-2-yl)-2,5-diphenyltetrazolium bromide (MTT) assay, adenosine triphosphate (ATP) assay, and resazurin assay, conducted to evaluate mitochondrial activity and overall cell health. Meanwhile, the lactate dehydrogenase (LDH) leakage assay and calcein-acetoxymethyl (Calcein-AM) assay have offered insights into membrane integrity and metabolic activity [44,48,51,54].

In most studies, oxidative stress caused by OPFRs was assessed by measuring reactive oxygen species (ROS) production using dichlorofluorescein-based substrates. The cell response to oxidative stress has been evaluated based on activation of the luciferase reporter gene nuclear factor erythroid 2-related factor 2 (Nrf2) [33,54], along with gene expression analysis and tetramethylrhodamine ethyl ester (TMRE) staining for mitochondrial membrane potential [49].

The genotoxicity of OPFRs has been investigated using single-cell gel electrophoresis (Comet assay), which detects DNA strand breaks, and Hoechst 33342 staining, which visualizes chromatin alterations and membrane damage [44,48].

The gene expression analysis of tumor protein 53 (p53), cyclin-dependent kinase inhibitor 1A (p21), and DNA damage-inducible beta (Gadd45β) was also evaluated for potential carcinogenic risks [34,49].

Also, the endocrine-disrupting behavior of OPFRs was investigated using luciferase reporter assays that evaluate OPFR interactions with hormone receptors, including estrogen receptor alpha (ERα) and mineralocorticoid receptor (MR) [28,32]. Additionally, steroidogenic gene expression analysis using quantitative reverse transcription polymerase chain reaction (qRT-PCR) has been used to understand the changes in testosterone and estradiol production [28,30,49]. To assess early differentiation disruption, various approaches have been employed, including differentiation assays using mouse embryonic stem cells [29]. Additionally, neuroinflammation and neuronal health were evaluated by analyzing neurite outgrowth and microglial activation through immunostaining and cytokine release assays [54].

As a very relevant part of NAMs, omics technologies, such as transcriptomics, proteomics, metabolomics, and lipidomics allow researchers to analyze molecular changes in response to chemical exposure, helping to identify the toxicity mechanisms at different levels of molecular organization. These techniques can detect subtle molecular changes that may not be observable with the traditional toxicity assays, improving the accuracy of risk assessments. In OPFR research, omics technologies have been used either alone or in combination (multi-omics) to investigate toxicity mechanisms. Most studies have focused on hepatotoxicity, utilizing normal human hepatocytes or liver hepatoma cells. Transcriptomics is the most commonly applied approach [70], sometimes combined with metabolomics [53,54,55], lipidomics [52], or even proteomics for a more comprehensive analysis [40,71,72,73]. Beyond the liver, transcriptomics and metabolomics have been used to assess neurotoxicity and immunotoxic effects in 3D brain spheres and THP-1 cells, respectively [42,60]. Additionally, transcriptomics and lipidomics have been applied to study OPFR toxicity in ovarian granulosa cells [31], while transcriptomics alone has been used to investigate toxicity in colon cancer cells [74].

Regarding computational methods, in silico molecular docking and quantitative structure–activity relationship (QSAR) modeling, have been employed. Also, in silico toxicokinetic and pharmacokinetic modeling, including multi-compartment Physiologically Based Kinetic (PBK) modeling has been employed to simulate OPFR distribution and concentrations in several organs, providing a reconstruction of the oral exposure that can be extrapolated to humans for toxicological risk assessments [50]. Risk assessment calculations, including the Hazard Quotient (HQ) and carcinogenic risk modeling are carried out to help understand the possible health risks linked to OPFR exposure [36].

## 3. Toxicological Studies of OPFRs in Vitro

This section provides a comprehensive overview of in vitro studies aimed at assessing the toxicological effects of OPFRs on various organ systems and understanding how OPFRs affect different tissues. To support these assessments, analyzing OPFR distribution in biological samples and the environment is necessary. Techniques, such as ultra-performance liquid chromatography coupled with high-resolution tandem mass spectrometry (UPLC-HRMS/MS), provide accurate measurements of OPFR concentrations in both biological and environmental samples.

### 3.1. Liver Toxicity

Testing the toxicity of OPFRs on liver cells is highly relevant due to the liver’s critical role in metabolizing and detoxifying xenobiotics (foreign substances). The potential induction of hepatotoxicity could not only impair detoxification processes but also disrupt metabolic homeostasis, including glucose regulation, lipid metabolism, and protein synthesis. Chemical-induced liver dysfunction could lead to widespread systemic effects, ultimately compromising overall health [75,76].

The effects of OPFRs on cell viability, DNA damage, oxidative stress, and the membrane integrity of OPFRs have been tested in HepG2 cells (Table S1). TMPP, TPhP, EHDPHP, and TDCIPP were non-cytotoxic at concentrations up to 10 µM but reduced mitochondrial, lysosomal, and esterase activity at 25 µM (EHDPHP, TPhP) [37]. In another study, TPhP, TBP, TBOEP, and TCPP were tested at 25, 50, 100, and 200 µM concentrations. The results showed cell mortality, oxidative stress, and DNA damage only at concentrations higher than 100 µM [44]. In primary cultures of chicken embryonic hepatocytes (CEHs), Crump et al. investigated the cytotoxic and molecular effects of TDCPP and TCPP [51]. Cell viability assays showed that TDCPP was cytotoxic at concentrations above 10 µM, with an LC_50_ of 60 µM, whereas TCPP exhibited no cytotoxicity up to 300 µM [51].

Concerning the genotoxic potential of OPFRs, a study of Li et al. integrated in vitro and in silico methods to examine the interaction of nine OPFRs, TCEP, TCPP, TPrP, DnBP, TEHP, TPhP, TEP, TMP, and TCP, with the p53 tumor suppressor gene, a key regulator of the DNA damage response and apoptosis [34]. Two OPFRs, TCPP and TPhP, induced the dose-dependent upregulation of p53 mRNA after exposure to 1 µM, 10 µM, and 100 µM, in human embryonic liver L02 cells, indicating that OPFRs can trigger DNA damage responses, potentially leading to apoptosis. In addition, by using fluorescence spectroscopy, robust DNA binding through competitive interactions was confirmed. Structural features, like the aromaticity and electrostatic potential, influenced the binding affinity. A QSAR model (R^2^ = 0.924, Q^2^ = 0.890) identified key factors influencing OPFR–DNA binding, which include the binding site count, molecular electrostatic potential, and electronegativity. OPFRs with a stronger negative electrostatic potential demonstrated higher DNA binding, potentially disrupting its structure and function [34].

In another study, activation of the aryl hydrocarbon receptor (AhR) by OPFRs was investigated using the reporter rat hepatoma cell line H4IIE-CALUX. AhR is a ligand-activated transcription factor that regulates the expression of various genes involved in detoxification. Ten of the eleven OPFRs tested activated AhR [33]. TCEP, TCIPP, TPhP, TIPPP, TMPP, and TEHP showed strong activation (EC50 ≤ 10 µM), with TMPP having the highest activation (340% efficacy). These results demonstrated that OPFR structural features are key to AhR activation. Aryl OPFRs (e.g., TPhP, TIPPP, TMPP) showed enhanced receptor activation, whereas chlorinated OPFRs (e.g., TDCIPP) demonstrated little or no activation, indicating that halogenation reduces the interaction. Conversely, another study demonstrated that TDCIPP induced stress responses (e.g., xenobiotic metabolism and ABC transporter pathways) at the transcriptional level after exposure to a subtoxic concentration (10 μM) [40]. In conclusion, AhR activation by OPFRs raises toxicological concerns, including alterations to liver enzyme induction, increased oxidative stress, and potential endocrine disruption.

LO2 normal hepatocytes have been widely used to study transcriptional changes induced by OPFR exposure. THP was found to alter pathways related to endoplasmic reticulum (ER) stress, apoptosis, cell cycle regulation, and glycolysis. TPhP triggered significant changes in pathways associated with apoptosis, oncogene activation, redox homeostasis, and DNA damage and repair [77]. EHDPhP notably disrupted energy homeostasis, ER stress, apoptosis, cell cycle, and inflammatory responses.

Transcriptomic studies using HepG2 hepatoma cells revealed that both TCEP and its analogue TCIPP affect genes involved in immune function regulation—primarily those encoding effector proteins in the complement cascade—as well as steroid hormone biosynthesis and xenobiotic metabolism pathways, exhibiting similar transcriptional effects [70]. Additionally, another study on TCEP toxicity in HepG2 cells identified significant alterations to genes associated with human cancer pathways, suggesting that TCEP may act as a cancer-inducing compound [73]. In line with this, a qPCR array in HepG2 cells showed that TEHP exposure upregulated 10 genes and downregulated 4 genes linked to human cancer pathways, further supporting its potential carcinogenic effects [78]. A similar pattern was observed in TDCPP-exposed HepG2 cells, where several cancer-related genes were significantly altered [79].

To determine the impact of OPFRs on lipid metabolism, TMPP, TPhP, EHDPHP, and TDCIPP were examined using HepG2 liver cells, molecular docking, and computational analyses [37]. At lower concentrations (2 µM and 10 µM), there was an increase in intracellular lipid droplets in a dose-dependent manner, suggesting disrupted lipid metabolism and potential hepatic steatosis even at low micromolar levels. TMPP and TPhP upregulated key lipogenic genes (SREBP-1c, DGAT2, SCD1), promoting fatty acid synthesis and triglyceride formation. In addition, TMPP, TPhP, EHDPHP, and TDCIPP bind strongly to key regulators of lipid metabolism, such as PXR and PPARγ, initiating events in hepatic steatosis. Conversely, mitochondrial dysfunction was observed, resulting in impaired ATP production. This dual disruption (lipid buildup and reduced energy) connects OPFR exposure to hepatic steatosis and poses public health concerns [37]. Consistent with this lipid metabolism disruption, a study using liver slices from Atlantic cod revealed that exposure to TCPP and EHDPHP disrupted the expression of key genes involved in cholesterol biosynthesis and lipid metabolism, as evidenced via RNA sequencing [80].

A study investigating seven OPFRs—TDCPP, TCEP, TCPP, TnBP, TBOEP, TPhP, and TOCP—using HEK-293 and HepaRG liver cell models provided detailed insights into their interactions with human drug transporters. The results showed that OPFRs selectively block specific transporters. Notably, TBOEP, TDCPP, TOCP, and TPhP blocked key transporters, including OAT3, OATP1B1, OATP1B3, OCT2, and BCRP, interfering with the transport of key endogenous compounds, such as estrone-3-sulfate, cholecystokinin octapeptide (CCK-8), and dopamine. These results suggested that OPFRs may disturb hormone signaling, neurotransmitter regulation, liver drug metabolism, and endogenous compound elimination, potentially altering physiological processes [43].

Regarding the biotransformation of OPFRs occurring in the liver, a study investigated the biotransformation and metabolic kinetics of the chlorinated OPFR TDCPP and the aryl OPFR TPhP using mouse liver microsomes in vitro. A metabolomic analysis showed that TDCPP has a higher potential for bioaccumulation than TPhP, as indicated by their metabolic rates and half-lives (TDCPP: 1.8083 h; TPhP: 0.1531 h). Enzyme inhibition assays identified CYP2E1, CYP2D6, CYP1A2, and CYP2C19 as key enzymes in the biotransformation of TDCPP, while CYP2E1 played an important role in the metabolism of TPhP [53].

Since OPFRs are not found alone in the environment, it is essential to study their effects in the context of other potentially toxic compounds. A study investigated the in vitro toxicity of complex chemical mixtures, including OPFRs (TnBP, TPhP, TCPP, EHDPHP, TBOEP, TDCIPP, and TEHP), phenols, perfluoroalkyl substances (PFAS), and heavy metals in HepG2 liver cells. Fifty mixtures, each containing seven to ten chemicals, were evaluated at equitoxic EC10 levels to examine the interaction effects. Cytotoxicity measurements were compared to predictions from the Concentration Addition (CA) model [39]. Among the mixtures, the most toxic combination contained three PFASs (PFOA, PFNA, PFHxS) and five OPFRs (TnBP, TPhP, TCPP, EHDPHP, and TBOEP), with an EC_10_ of 112.5 µM. The least toxic mixture included PFHxS, 2,5-DCP, three OPFRs (TPhP, TCPP, TEHP), and three heavy metals (lead chloride, cobalt chloride, cadmium chloride), with an EC_10_ of 510.0 µM. Although there are some differences in cytotoxicity, all EC_10_ values for the mixtures are notably higher than the usual plasma concentrations seen in human biomonitoring studies [39].

### 3.2. Neurotoxicity

In vitro research on neuronal cell lines (PC12, SH-SY5Y) revealed concentration-dependent cytotoxicity for TDCPP (tested at 50 µM and 2.5–20 µM, respectively) and TCEP (tested at 200 µM), primarily through pathways of MAPK (Mitogen-Activated Protein Kinase) and CaMK2 (Calcium/Calmodulin-Dependent Protein Kinase II) [50,54]. These exposures caused cell death through apoptosis and altered the expression of neurodevelopmental genes [50,54] (Table S2).

A physiologically based kinetic (PBK) model demonstrated that TDCPP, TCIPP, and TCEP were metabolized into bis(1,3-dichloro-2-propyl) phosphate (BDCIPP), bis(1-chloropropyl) phosphate (BCIPP), and bis(2-chloroethyl) phosphate (BCEP), respectively. These metabolites acted as key biomarkers for exposure to OPFRs, and they had been detected in human urine, plasma, and breast milk, raising concerns about their neurotoxic, carcinogenic, and systemic effects. The bioaccumulation of TDCIPP, TCIPP, and TCEP was observed in brain tissues, with slow elimination over time, highlighting the potential risks of long-term exposure and accumulation in the nervous system [50]. TDCIPP caused cytotoxic effects in neuronal cells at concentrations >10 µM, with LC_50_ values of ~28.7 µM, while TCPP was not cytotoxic up to 300 µM [51].

Oxidative stress and inflammation have been shown in the neurotoxicity studies of OPFRs. Inflammatory responses, marked by high cytokine levels, such as IL-1β and TNFα, were observed in neuronal cultures and animal studies [55]. Nrf2 activation, a marker of oxidative stress, was noted in some studies, with TDCPP increasing Nrf2 expression at 10 µM in SH-SY5Y cells [54].

Reduced nerve growth and changes in key genes, like BDNF, disrupted neural flexibility, with these effects observed in various neuronal models exposed to TDCIPP and TPhP at concentrations ranging from 0.003 µM to 30 µM. Human-derived neural models exhibited greater sensitivity to OPFRs than rodent models, showing the importance of human-based studies [54].

Further evidence of OPFR-induced neurotoxicity was observed in PC12 cells exposed to TDCIPP. This OPFR was found to significantly alter the circRNA expression profile. CircRNAs are circular noncoding RNAs that participate in a variety of biological functions in vivo. Specifically in the brain, circRNAs are highly enriched and are key regulators of neuronal function, impacting gene expression, synaptic activity, and neuroprotection. Furthermore, analyzing circRNA microarray data from PC12 cells exposed to TDCIPP, there were 3432 differentially expressed circRNAs (*p* < 0.05), with 1682 increased and 1750 decreased. KEGG pathway analyses suggested that rno_circRNA_013845, rno-miR-361-3p, and rno-miR-702-3p may be involved in the regulation of Traf2 expression, influencing the NF-κB signaling pathway and promoting apoptosis [57].

Another significant mechanism of OPFR-induced neurotoxicity involves the inhibition of O-linked N-acetylglucosamine transferase (OGT), an enzyme crucial in brain function due to its involvement in neuronal signaling, neuroprotection, synaptic plasticity, and memory formation.

In 12 tested OPFRs, six with aromatic or chlorinated alkyl groups significantly inhibited OGT activity, with TCrP being the strongest. Molecular docking showed that the inhibitory effects may be changed based on the structure. Since OGT is essential for maintaining neuronal health, its dysregulation can contribute to neurodevelopmental disorders. These OPFRs also decreased protein O-GlcN acylation in PC12 cells and induced ROS, calcium levels, cell proliferation, and autophagy [41].

To enhance the physiological relevance of toxicological conclusions, a recent study employed a 3D rat brain organotypic model to compare the neurotoxic effects of BDE-47, a widely used PBDE, with four OPFRs: IPP, TPhP, IDDP, and TMPP [42]. Metabolomic and transcriptomic analyses revealed stronger neurotoxic effects of OPFRs at human-relevant, non-cytotoxic levels (0.1–5 µM). OPFRs caused neuronal toxicity in the low µM range and, unlike BDE-47 and TPhP, reduced glutamate and GABA neurotransmitter levels. Additionally, all examined OPFRs decreased N-acetyl aspartate (NAA), a key biomarker of neuronal health, and downregulated plasma membrane dopamine transporter expression. These results also indicated the induction of astrogliosis and an inflammatory response. Pathway analysis further revealed disruptions in action potential transmission, synaptic signaling, the immune response, cell cycle regulation, and lipid metabolism. Collectively, these findings suggested that the investigated OPFRs induced a more severe neurotoxic phenotype than the progressively substituted brominated flame retardant BDE-47 [42].

### 3.3. Endocrine Disruption

OPFRs have been identified as endocrine disruptors because of their capability to block or modify hormone activity through interactions with nuclear receptors, including estrogen receptors (ERs), androgen receptors (ARs), and glucocorticoid receptors (GRs) (Table S3).

Deepika et al. investigated the endocrine-disrupting effects of the OPFR metabolites BCIPP, BDCIPP, and DPHP [50]. These metabolites originate from the parent OPFRs TCIPP, TDCIPP, and TPhP, respectively. The obtained results indicated that biotransformation could increase the toxicity of OPFRs, as some metabolites showed stronger endocrine-disrupting effects than their parent compounds. The research employed in vitro assays with mammalian cell lines (CHO-K1 cells and H295R cells) to evaluate receptor activity and employed zebrafish embryos to assess developmental toxicity at three concentrations: 10⁻^7^ M, 10⁻^6^ M, and 10⁻^5^ M. BCIPP demonstrated the strongest estrogen-like activity as an estrogen receptor alpha (ERα) agonist, with a 6.5 × 10⁻^8^ M of REC_20_ (Relative Estrogenic Concentration 20%) to activate the estrogen receptor. BDCIPP was most effective as a mineralocorticoid receptor (MR) antagonist, with a 1.1 × 10⁻^8^ M of RIC_20_ (Relative Inhibitory Concentration 20%) to block MR signaling. DPHP exhibited weaker endocrine-disrupting effects compared to the other two metabolites. The results showed that OPFR metabolites interfere with hormonal receptors by mimicking hormones or blocking their functions, which increases health risks. Studies on zebrafish embryos revealed that the highest concentrations of these metabolites caused physical malformations, like pericardial and yolk sac edema and body curvature. A gene analysis showed significant disruptions in stress and reproductive hormone pathways (HPA and HPG axes), with increased activity in genes like *ESR1* (coding for ERα), *CYP17*, and *CYP19b*. BCIPP was associated with the highest mortality rate, followed by BDCIPP and DPHP [28]. These results show that OPFRs and their metabolites significantly disrupt endocrine systems, with metabolites often being stronger than their parent compounds.

In another study, 11 OPFRs, including TMP, TEP, TPrP, TBP, TCPP, TCEP, TBOEP, TDCPP, TEHP, TPhP, and TCP, were analyzed for their interactions with human nuclear receptors, including (ERα), ERβ, androgen receptor (AR), glucocorticoid receptor (GR), thyroid hormone receptor α1 (TRα1), TRβ1, retinoic acid receptor α (RARα), retinoid X receptor α (RXRα), pregnane X receptor (PXR), peroxisome proliferator-activated receptor α (PPARα), and PPARγ [47]. This study, carried out in ovarian hamster CHO-K1 and simian kidney COS-7 cell lines at 1.2 to 24 µM OPFRs, revealed various effects on nuclear receptors. TPhP and TCP showed weak estrogen-like activity but stronger anti-androgenic effects, while TPhP and TDCPP activated PXR, potentially altering detoxification enzyme expression. Most OPFRs were not highly toxic within the tested range, though TEHP and TCP slightly reduced cell viability at higher doses. Aromatic OPFRs with phenyl rings bonded more strongly to hormone receptors, highlighting the influence of their structure on endocrine disruption [47].

In a study by Rosenmai et al., exposure to OPFRs in the H295R adrenocortical carcinoma cell line disrupted key genes involved in steroid hormone production, leading to endocrine effects [33]. Tested OPFR concentrations ranged from 12.5 to 100 μM. TCEP dropped CYP19A1 levels, which may reduce estrogen production. TPhP and TDCIPP increased CYP19A1 at high doses, suggesting estrogen-like effects. These changes impacted hormone levels: testosterone was increased with TCEP, TPhP, and TDCIPP, while estradiol was decreased with TPhP and, to a lesser extent, with TCIPP and TDCIPP. Consistent with the previous study, the more hydrophobic compounds, such as TPhP, exhibited stronger endocrine effects.

Two studies investigated the impact of oxidative stress pathways and estrogen-related mechanisms on breast cancer cells. In one study, the authors used AREc32 cells, a modified MCF7 human breast cancer cell line, to test whether OPFRs cause oxidative stress by activating the Nrf2 pathway. To investigate this, ten different OPFRs were tested at concentrations ranging from 12.5 to 100 µM for 24 h. The results showed that none of the tested OPFRs significantly activated the Nrf2 pathway, indicating that oxidative stress was not a major cause of toxicity for these OPFRs under the tested conditions [33].

Also, in the breast cancer context, another study using MCF7 cells investigated the estrogen-disrupting effects of 14 OPFRs categorized based on their chemical structure into alkyl-OPFRs (TMP, TEP, TPhP, TnBP, TiBP, THP) and aryl-OPFRs (MPhP, DPK, MDPP, TPhP, CDP, TCP, IPPDP, IDPP). The assay results showed that only TPhP demonstrated ERα-mediated agonistic activity, whereas most OPFRs, except TMP, induced proliferation in MCF7 breast cancer cells at higher nanomolar to micromolar concentrations [32].

### 3.4. Reproductive and Developmental Toxicity

Although the reproductive and endocrine systems are closely linked, this section focuses specifically to the studies investigating the impact of OPFRs on fertility and embryo development (Table S4).

Two similar studies by Feng et al. were carried out to investigate the effects of TDCIPP and TPhP on the progress of sperm growth and development, by using the mouse spermatocyte GC-2 cells [58,61]. In one study, cells were exposed to TDCIPP at concentrations of 1, 3, 10, and 30 μM for 48 h, with cell viability evaluated over up to 100 μM at 24, 48, and 72 h. The LC_50_ values decreased over time, demonstrating dose- and time-dependent cytotoxicity. Significant effects were observed at concentrations of 30 μM or higher. The assays showed that TDCIPP exposure induced apoptosis, with caspase-9 activity increasing by 87.5% and caspase-3 by 104.2% at 30 μM. Mitochondrial dysfunction was indicated by fragmentation, decreased ATP levels (31.3% at 30 μM), and altered mitochondrial membrane potential (MMP). ER stress was also apparent and characterized by upregulated Bip, CHOP, and ATF4 gene expression. These findings suggest that TDCIPP-induced cytotoxicity, mitochondrial dysfunction, and ER stress could harm male reproductive health by disrupting cellular homeostasis [58].

The other study of Feng et al. focused on uncovering the toxicological mechanisms associated with exposure to TPhP, also in spermatocyte GC-2 cells [61]. Exposure to TPhP significantly reduced cell viability, with sharp viability declines at concentrations higher than 30 µM. Oxidative stress, mitochondrial damage, reduced DNA integrity, and apoptosis were confirmed at 30 µM and 60 µM, indicating compromised genomic stability and potential harm to reproductive health. Although the tested concentrations exceed environmental levels, the findings underscore the risks associated with high or prolonged TPhP exposure [61].

Furthermore, another study examined the effects of seven commonly used OPFRs (EHDPHP, IDDP, TPhP, BPDP, IPPP, TMPP, and TOCP) compared to 2,2′,4,4′-tetrabromodiphenyl ether (BDE-47), a brominated flame retardant—on MA-10 mouse Leydig cells. These cells, originated from a Leydig cell tumor in the testis, are widely used in research to study testicular functions. The research focused on their impact on mitochondrial activity, cell survival, oxidative stress, steroid secretion, and gene expression [30]. Using concentrations ranging from 1 to 100 µM over 48 h, the study revealed that OPFRs were more cytotoxic than BDE-47. Most OPFRs compromised mitochondrial activity at 20 µM, whereas BDE-47 required at least a 50 µM concentration. IPPP demonstrated the highest cytotoxicity (IC_50_: 12.4 µM for mitochondrial activity; 10.3 µM for cell counts), while TPhP exhibited the least toxicity (IC_50_: 38.3 µM and 27.5 µM, respectively). All OPFRs induced oxidative stress at 10 µM, with IPPP being the most potent and TPhP being the least.

OPFRs also disrupted steroidogenesis. Unlike TPhP and BDE-47, the OPFRs EDHP, IDDP, BPDP, and TMPP elevated progesterone secretion by up to three times at 10 µM, while IPPP amplified dbcAMP-stimulated secretion by approximately two times. TOCP reduced LH-stimulated secretion, suggesting interference with receptor pathways. Also, key steroidogenic genes were also impacted. These results highlighted the significant risks on testicular function posed by OPFRs, which occurred at lower concentrations than BDE-47 [30].

Regarding the effects on female reproduction, a study by Zhang et al. investigated the in vitro exposure of mouse oocytes to EHDPP and compared the impact with the protective role of melatonin, a natural antioxidant [81]. EHDPP interfered with oocyte maturation, stopping at the metaphase I (MI) stage, caused cytoskeletal damage, and increased ROS levels. scRNA-seq analysis showed that EHDPP primarily impaired mitochondrial function, kinetochore-microtubule (K-MT) attachment, DNA damage, apoptosis, and histone modification, all improved by melatonin [81].

Moreover, Wang et al. studied the effects of OPFR mixtures on ovarian granulosa cells, which play essential roles in female reproduction [31]. After exposure to three OPFR mixtures at environmentally relevant doses (0.0009 µg/mL to 888 µg/mL) found in dust houses, many phenotypic and functional endpoints were affected in granulosa cells. Transcriptomic and lipidomic analyses revealed alterations in cholesterol synthesis and lipid metabolism pathways. This study provided novel evidence that a realistic exposure to OPFRs found in house dust can impact the structure and function of ovarian granulosa cells. Further research is needed to decipher the mechanisms of OPFR-induced toxicity and the potential impact of the changes induced on female reproductive function.

To explore the gestational effects of OPFRs, the study of Zhang et al. explored the impact of EHDPHP on placental function and highlighted the protective role of LXRα (Liver X Receptor Alpha) in alleviating its harmful effects [35]. LXRα is a nuclear receptor that plays a crucial role in regulating lipid metabolism, cholesterol homeostasis, and inflammation, all of which are essential in placental development. EHDPHP was administered at concentrations of 10, 20, and 30 μM in HTR-8/Svneo cells (human extravillous trophoblasts from a first-trimester placenta) and JEG-3 cells (a human choriocarcinoma cell line), leading to a dose-dependent reduction in cell viability, migration, and angiogenesis. In animal studies, pregnant ICR mice were given 0.4, 2, and 10 mg/kg/day of EHDPHP from gestational days 7.5 to 17.5, with higher doses negatively impacting placental size and fetal growth by disrupting essential biological functions. In both models, EHDPHP increased inflammatory cytokines (IL-6, IL-1β, and TNF-α), linking inflammation to placental dysfunction and adverse pregnancy outcomes. At lower doses (10 μM in vitro, 0.4 mg/kg/day in vivo), EHDPHP caused moderate effects, such as slight disruptions in migration and angiogenesis. However, severe toxic effects, including inflammation and significant cell damage, were observed at higher concentrations (30 μM in vitro, 10 mg/kg/day in vivo) [35].

In another study, TPhP, IPP, and TDCIPP were tested in mouse embryonic stem cells, in a range of concentrations from 0.003 μM to 100 μM, to determine the point of departure (POD) for specific effects [29]. The results showed cytotoxicity in the 1–10 μM range, along with low levels of Goosecoid (GSC), a key marker of embryonic development, indicating disrupted embryonic differentiation. Cytotoxicity and decreased GSC expression were observed at relatively high concentrations, with POD values of 41 μM for TPhP, 66 μM for IPP, and 44 μM for TDCIPP [29].

Another study examined EHDPHP and IDDPP, focusing on their effects on amniogenesis in human amniotic sac embryoids as an amniogenesis disruption could lead to miscarriage. A high-throughput assay screened 53 OPFRs for their effects on OCT4 expression, a fundamental transcription factor in early embryonic development, in human embryonic stem cells. EHDPP and IDDPP exhibited the most significant inhibition, reducing OCT4 levels to 50% and 53% of the control, respectively. Testing OPFRs on in vitro amniogenesis models indicated disruptions in the process, likely through inhibition of the ITGβ1 pathway, providing evidence of their potential association with biochemical miscarriage [38].

Finally, Zhang et al. evaluated the early embryonic developmental toxicity of neonicotinoids (NEOs) and OPFRs by exposing differentiating human embryonic stems cells to three NEOs and three OPRFs (TEP, TPhP, and TCIPP) at 1 μM for eight days [59]. A transcriptomic analysis revealed that both NEOs and OPFRs activated the BMP4 signaling pathway, which is crucial for early differentiation and pluripotency maintenance, suggesting that these compounds may impact early human development.

### 3.5. Lung Toxicity

OPFRs are commonly found in particulate matter (PM) suspended in the air and pose health risks in outdoor and indoor environments (Table S5). To investigate the effect of OPFRs found outdoor, a study reported the analysis of a total of 11 OPFRs in PM_2.5_ samples from two Chinese cities and grouped based on their structures, including chlorinated OPFRs (TCEP, TCPP, and TDCPP), aryl-OPFRs (TPhP, EHDPHP, and TCP), and alkyl-OPFRs (TEP, TiBP, TnBP, TBOEP, and TEHP) [36]. To investigate these OPFRs’ toxicity, human bronchial epithelial cells (BEAS-2B) were exposed to PM_2.5_ extracts. Decreased cell viability and increased oxidative stress based on reactive oxygen species (ROS) production were observed, indicating dose-dependent cytotoxic effects and potential respiratory health impacts. A health risk assessment found that toddlers’ estimated daily intake (EDI) of OPFRs ranged from 0.07 to 39.9 ng/kg/day, while adults’ intake was lower, ranging from 0.03 to 14.0 ng/kg/day. Although all groups had Hazard Quotients (HQ) below 1—suggesting no immediate health risks—the study raised concerns about long-term exposure [36].

Yuan and coworkers explored the toxic effects of chlorinated OPFRs, specifically TCEP and TCPP, focusing on their potential to cause cell damage [49]. This study tested these two compounds in A549 lung epithelial cells at concentrations from 0.1 μM to 1000 μM, with significant effects observed at 50 μM and 100 μM. Both compounds share pathways related to oxidative stress and mitochondrial dysfunction. However, TCPP exhibited stronger effects, notably through robust activation of the p53 pathway, leading to DNA damage, cell cycle arrest, and apoptosis [49].

In addition, An et al. evaluated the impact of four OPFRs—TPhP, TBP, TBOEP, and TCPP—on A549 cells [44], in a range of concentrations from 25 to 200 µM. All OPFRs induced a concentration-dependent decrease in cell viability, the induction of oxidative stress (3.8-fold), DNA damage (1.7-fold), and cellular membrane disruption (1.2-fold). Among the tested OPFRs, TPhP and TBOEP were especially harmful, underscoring the need to assess their safety profiles further [44].

Finally, a recent study conducted by our research group examined the effects of seven OPFRs (TBOEP, TPhP, EHDPhP, TDCPP, TEHP, TCP, and TCEP) also on A549 lung cells using both 2D and 3D culture models (data pending publication). Cells were exposed to 25, 50, and 100 μM OPFRs for 72 h. The results revealed dose-dependent toxicity, with TPhP and TDCPP significantly reducing cell viability at 100 μM. A lipidomic analysis identified significant disruptions in lipid metabolism, particularly in triacylglycerols (TGs), diacylglycerols (DGs), ceramides (Cers), phosphatidylinositol (PI), and ether-linked phosphatidylethanolamine (PE-O). Additionally, the accumulation of TG, indicative of increased energy storage, was further confirmed based on the formation of lipid droplets. The results of this study suggested the disruption of key toxicological pathways, including oxidative stress, inflammatory signaling (IL-8 upregulation), and apoptosis (ceramide accumulation). These pathways are implicated in lung diseases, such as COPD and fibrosis. Furthermore, OPFRs containing aromatic rings (TPhP, EHDPhP, TCP) or multiple chlorine atoms (TDCPP) showed stronger toxicity, whereas those with aliphatic structures (TEHP, TCEP) had milder effects.

### 3.6. Other Tissues

The toxicity of OPFRs in other tissues has also been assessed using various cell types, including colon cancer cells, peripheral blood mononuclear cells, immune cells, corneal epithelial cells, and vein epithelial cells (Table S6).

The effects on the intestinal barrier were explored using Caco-2 cells, a human epithelial cell line derived from colorectal adenocarcinoma. This cell line was exposed to TPhP, TBP, TBEOP, and TCPP at concentrations of 25, 50, 100, and 200 μM. Significant toxicity was observed at 100 and 200 μM, DNA damage was high at these concentrations, and membrane integrity was affected. However, ROS levels did not significantly increase, indicating the toxicity was not related to oxidative stress. These results highlighted the potential risks of OPFRs to intestinal health, indicating that these chemicals may compromise gut epithelial integrity due to their toxicity in Caco-2 cells [44].

Another study investigated the effects of TDCPP, TCEP, and TCPP on the human intestinal flora and Caco-2 cells [56]. TDCPP exposure changed the community structure of the intestinal microbiota, leading to a gradual increase in opportunistic infections. The exposure also inhibited carbohydrate metabolism pathways in gut bacteria, favoring them to develop increased resistance mechanisms. All three organophosphorus flame retardants negatively affected Caco-2 human colorectal adenocarcinoma cells. Among them, TDCPP showed the strongest toxicity, by disrupting cellular homeostasis at 10 μM. The combined effects of TDCPP on both intestinal cells and the gut microbiota are likely to exacerbate intestinal health issues.

Mokra et al. investigated the in vitro cytotoxic effects of TCEP and TCPP on peripheral blood mononuclear cells (PBMCs) [48]. The results showed that TCPP was more toxic than TCEP. TCPP significantly reduced cell viability at 0.5 mM and higher, dropping to 47.7% at 1 mM. On the contrary, TCEP only reduced cell survival to 76.9% at 1 mM. These differences may have been caused by their structure, as TCPP’s methyl groups made it more reactive to affecting cell functions. Morphological changes provided additional evidence that TCPP is more cytotoxic. PBMCs exposed to TCPP displayed cell shrinkage, increased granularity, and disrupted membrane integrity at 0.25 mM. In contrast, TCEP-induced changes were minimal and became significant only at the highest tested concentration. These results were confirmed via fluorescence microscopy, showing that TCPP caused severe DNA condensation and cell membrane damage at higher levels, while TCEP had weaker effects [45]. The study concluded that TCPP is significantly more cytotoxic than TCEP, leading to increased cell damage and reduced viability in PBMCs [48].

Regarding the impact on the immune system, the effects of TPhP and TOCP were investigated using THP-1 macrophages. Transcriptomic and metabolomic analyses revealed that both compounds caused similar disruptions, including impairments in nucleic acid synthesis and energy metabolism, leading to reduced ATP levels. However, an integrative analysis uncovered opposing immunotoxic effects. TPhP inhibited phagocytosis and adhesion, while TOCP activated mTOR signaling, enhancing immune responses. These results highlighted the distinct immunomodulatory mechanisms of TPhP and TOCP, paving the way for further research into the effects of structurally diverse OPFRs [60].

Furthermore, human corneal epithelial cells (HCECs) were exposed to TPP to assess cell viability, morphology, apoptosis, mitochondrial membrane potential, and the underlying mechanisms. TPP reduced cell viability with an IC_50_ of 220 μM. The results indicated that TPP induced apoptosis, as evidenced by increased apoptosis levels, a dose-dependent reduction in mitochondrial membrane potential, and altered expression of apoptosis-related genes, including Cyt c, Caspase-9, Caspase-3, Bcl-2, and Bax. These results may provide the basis for evaluating the potential impact of OPFRs on corneal health [62].

To investigate vascular toxicity, Saquib et al. exposed human umbilical vein endothelial cells (HUVECs) to TCPP at concentrations ranging from 5 to 400 μM for 24 h [52]. The results showed a significant reduction in cell survival at 200 and 400 μM, DNA damage at concentrations from 50 to 400 μM, increased ROS levels by 1.1- and 1.4-fold, and mitochondrial dysfunction increased by 1.16- and 1.48-fold. Also, cell cycle alterations were observed with 5.1% to 58.8% of cells entering the SubG1 apoptotic phase at TCPP concentrations between 5 to 400 μM [52]. All of these results indicate that TCPP is a genotoxic and pro-apoptotic agent, potentially eliciting similar toxic responses in the human vascular system.

## 4. Discussion and Future Perspectives

**Figure 2:**
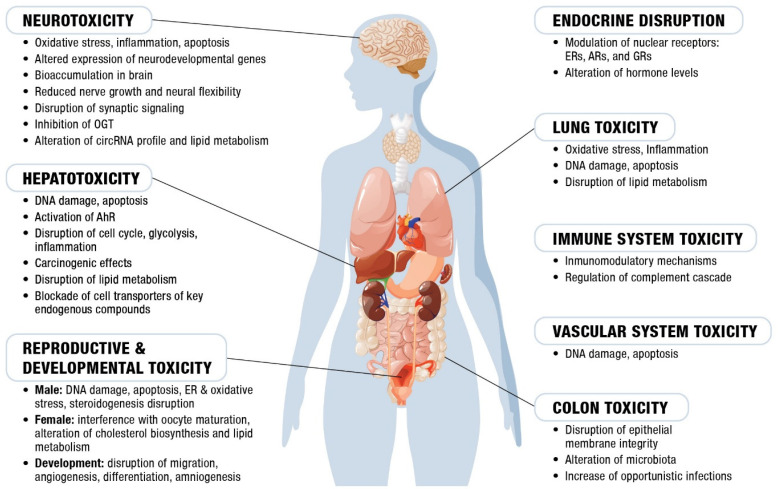
The main toxic effects of OPFRs reported in the reviewed studies, classified by the organs and biological systems affected. For detailed information on the OPFR species, concentrations used, and specific findings, refer to the Supplementary Tables S1–S6. AhR: hydrocarbon receptor; OGT: O-linked N-acetylglucosamine transferase; ER: estrogen receptors; AR, androgen receptors; and GR: glucocorticoid receptors.

The widespread presence of OPFRs in everyday products raises concerns about the potential impact of the daily exposure to these compounds on human well-being. The studies reviewed here have identified the severe toxic effects of OPFRs on various human organs and physiological functions using a range of NAMs, suggesting that exposure to these substances may pose significant risks to human health (Figure 2).

Most studies report a dose-dependent OPFR toxicity within the tens-to-hundreds micromolar range. In many cases, mitochondrial dysfunction, DNA damage, and apoptosis have been identified as common cell death mechanisms. Additionally, the activation of AhR and oxidative stress have been implicated in liver, neurological, and reproductive toxicity studies.

Interestingly, endocrine disruption and neurotoxicity have been observed at submicromolar concentrations, suggesting that certain biological systems are particularly sensitive to OPFR exposure. The application of omics technologies, especially transcriptomics (the most widely NAM used in OPFR studies) has revealed that even at subtoxic concentrations, significant gene expression changes occur. This indicates that molecular alterations take place at low doses, even in the absence of detectable effects on cell viability and phenotypes.

As previously mentioned, OPFRs can be categorized into chlorinated, alkyl, and aryl compounds, and their chemical structure can influence bioaccumulation, metabolism, and overall toxicity. For instance, aryl OPFRs (e.g., TPhP) contain aromatic rings that show strong endocrine-disrupting effects by acting as agonists of hormone receptors and impacting their functions. They also activate the antioxidant xenobiotic response through interactions with the AhR. Chlorinated OPFRs are more water-soluble and show reduced interactions with AhR receptor but tend to bioaccumulate. For instance, TDCPP, TCIPP, and TCEP have accumulated in brain tissues [50], due to lower metabolic rates and increased half-lives [53].

A critical issue is that the metabolic transformation of OPFRs is often associated with increased toxicity and stronger endocrine-disrupting effects, raising concerns about long-term health risks. This is evident when considering the biotransformation products BCIPP and BDCIPP (from TCIPP and TDCIPP), which showed enhanced interactions with ERα and MR receptors [28]. In addition, metabolites like BDCIPP and BCIPP have been detected in human urine and plasma, indicating the importance of considering OPFR metabolic transformations in health risk assessments [50].

Although in vitro studies provide valuable insights into OPFR toxicity, they also have notable limitations. While highly controlled, accurate, and reproducible, in vitro studies frequently fail to capture the complexities of whole organisms’ interactions. This limitation may result in inaccurate assessments, potentially underestimating or overestimating actual human health risks. In this context, integrating more physiologically relevant models, such as three-dimensional (3D) cultures, co-culture systems, or organ-on-chip technologies, can enhance the predictive power of in vitro assays by better simulating in vivo conditions. Furthermore, exposure duration is a key factor, due to the continuous exposure of OPFRs in outdoor and indoor environments and their potential bioaccumulation in tissues. To address this challenge, the use of advanced 3D cell culture models, such as organoids, could offer a solution by allowing for prolonged exposure experiments that better mimic real-life conditions and provide a scenario where understanding the toxic effects of a long term and low dose could be more feasible.

These studies are usually performed at concentrations ranging from micromolar to millimolar levels over a short period, simulating a short-term exposure, providing valuable insights into key toxicity mechanisms and helping elucidate the potential health risks associated with bioaccumulation. However, these concentrations exceed environmental levels and may not represent the accurate effects of chronic (long-term) low-dose exposure effects. As shown in many of the reviewed studies, omics methods, as NAMs, focused on increasing the analytical capabilities, enable the detection of molecular changes at very low doses. This increased analytical sensitivity underscores the need to apply these techniques to assess OPFR toxicity at environmentally relevant concentrations. Integrating multiple omics approaches (multi-omics) offers a powerful strategy to link different layers of molecular organization and deepen our understanding of OPFR toxicity mechanisms.

In addition, the more computationally intensive NAMs are mainly related to in silico pharmacokinetic modeling approaches, which provide effective solutions by converting in vitro data into realistic exposure scenarios considering OPFR absorption, distribution, and metabolism.

The use of NAMs in the REACH regulatory processes is generally supported, as the REACH Regulation (EC No. 1907/2006) encourages their application when appropriate for assessing the toxicity of industrial chemicals [82]. This support aligns with the increasing interest in NAMs, which is driven by concerns about animal welfare and the principles of the 3Rs: reduction, refinement, and replacement of animal testing. Formal evaluation systems have been developed in the United States and Europe to ensure that NAMs are scientifically valid and appropriate for regulatory use [83,84]. REACH’s commitment to these alternatives is reflected in its acceptance of specific in vitro assays [85], such as those for skin corrosion and serious eye damage, as substitutes for traditional in vivo tests. Furthermore, by aligning with OECD guidelines, REACH has established criteria for assessing NAMs, focusing on the accuracy, reproducibility, transparency, applicability, and mechanistic relevance [86].

Future research on OPFR toxicity should prioritize long-term exposure models and environmentally relevant concentrations to improve risk assessments and better predict health threats in real-life scenarios. By bridging the gap between NAM-based results and actual exposure scenarios, these innovative approaches could play a key role in shaping future policies designed to minimize the hazards associated with OPFRs.

## Figures and Tables

**Table 1:** List of the organophosphate flame retardants (OPFRs) referenced in this study, their full chemical names, and corresponding citations.

Acronym	Full Name	References
BCIPP	Bis(2-chloroisopropyl) phosphate	[28]
BDCIPP	Bis(dichloropropyl) phosphate	[28]
BPDP	Bisphenol diphenyl phosphate	[29,30,31]
CDP	Cresyl diphenyl phosphate	[31,32,33]
DnBP	Di-n-butyl phosphate	[34]
DPHP	Diphenyl phosphate	[28,33,35]
EDHP	Ethyldiphenyl phosphate	[30]
EHDPHP	Ethylhexyl diphenyl phosphate	[29,31,33,35,36,37,38,39,40,41]
IDDP	Isodecyldiphenyl phosphate	[29,30,32,38,42]
IPP	Isopropylphenyl phosphate	[29,30,31,42]
IPPDP	Isopropylphenyl diphenyl phosphate	[32]
MDPP	Methyl diphenyl phosphate	[32]
MPhP	Methylphenyl phosphate	[32]
TBOEP	Tris(butoxyethyl) phosphate	[31,36,37,39,40,41,43,44,45,46,47]
TBPP	Tris(2,3-dibromopropyl) phosphate	[45]
TCEP	Tris(2-chloroethyl) phosphate	[29,31,33,34,36,37,40,41,43,45,47,48,49]
TCIPP	Tris(2-chloroisopropyl) phosphate	[31,33,37,50]
TCP	Tricresyl phosphate	[32,36,40,41,47]
TCPP	Tris(2-chloropropyl) phosphate	[34,36,39,41,43,44,45,47,48,49,51,52]
TDCPP TDCIPP	Tris(1,3-dichloro-2-propyl) phosphate Tris 1,3-dichloroisopropyl) phosphate	[29,31,33,37,39,43,47,50,51,53,54,55,56,57,58,59]
TEHP	Tris(2-ethylhexyl) phosphate	[29,31,33,36,37,39,43,47,50,51,53,54,55,56,57,58]
TEP	Triethyl phosphate	[32,36,41,46,47,60]
TiBP	Triisobutyl phosphate	[32,36,46]
TIPPP	Tris(isopropylphenyl) phosphate	[33]
TMP	Trimethyl phosphate	[32,34,41,46,47]
TMPP	Trimethylphenyl phosphate	[29,30,31,33,37,42]
TnBP	Tri-n-butyl phosphate	[31,32,36,37,39,41,43]
TOCP	Tris(orthocresyl) phosphate	[30,43,61]
TPhP	Triphenyl phosphate	[29,30,31,32,33,34,36,37,39,40,41,42,43,44,45,47,50,53,54,60,62,63]
TPrP	Tripropyl phosphate	[41,47]

## Data Availability

No new data were created or analyzed in this study. Data sharing is not applicable to this article.

## Supplementary Materials

The Supplementary Table can be downloaded at: https://www.mdpi.com/article/10.3390/toxics13040297/s1. Table S1: Liver toxicity; Table S2: Neurotoxicity; Table S3: Endocrine disruption; Table S4: Reproductive and developmental toxicity; Table S5: Lung toxicity; Table S6: Other tissues.
