# Supplementary material for: Toxicity Assessment of Organophosphate Flame Retardants Using New Approach Methodologies
Source: Toxics. 2025 Apr 11;13(4):297. doi: 10.3390/toxics13040297 (PMC12031142; doi:10.3390/toxics13040297)
Supplement: Supplementary file 1 [file toxics-13-00297-s001.zip › toxics-3562198-supplementary.pdf]

Supporting Tables for:

Toxicity Assessment of Organophosphate Flame Retardants using New Approach Methodologies

*Maryam Pyambri, Joaquim Jaumot, Carmen Bedia**

Environmental Chemistry Department. Institute of Environmental Assessment and Water Research (IDAEA-CSIC), Jordi Girona 18-26, 08034, Barcelona, Spain

Keywords: organophosphate flame retardants, new approach methodologies, *in vitro* methods, cell cultures, omics, toxicity

Supporting Tables index

S1. Liver toxicity

S2. Neurotoxicity

S3. Endocrine disruption

S4. Reproductive and developmental toxicity

S5. Lung toxicity

S6. Other tissues

References

S1. Liver toxicity

**Cellular model**	Organism	OPFRs used	Concentration	Main results	Ref
HepaRG (hepatocellular carcinoma)	Human	TDCPP, TCEP, TCPP, TNBP, TBOEP, TPHP, TOCP	2 µM to 100 µM	• OPFRs selectively inhibited transporters like OCT2, OATP1B1, and OAT3.<br>• Impaired transport of hormones and neurotransmitters was observed.<br>• OPFRs downregulated transporter gene expression.<br>• IC50 values for inhibition reached as low as 6.1 µM.	[43]
Rat hepatoma (H4IIE-CALUX) (rat hepatoma cells)	Rat	TCEP, TCIPP, TDCIPP, TPHP, DPHP, CDP, TIPPP, TMPP, EHDPP, TEHP	0.4 µM to 100 µM	• All OPFRs activated the AhR pathway except TDCIPP.<br>• TMPP and CDP exhibited high efficacy (greater than 250%).<br>• Chlorinated OPFRs, including TDCIPP, showed minimal activation.<br>• AhR activation was dependent on concentration.<br>• This activation suggested potential endocrine-disruptive effects.	[33]
LO2 (human liver cells) and, AML12 (mouse hepatocyte cells)	Human<br>Mouse	THP, TiBP, TMP, TEP, TBOEP, TEHP	0 to 100 µg/mL, 400–800 mg/kg	• THP-induced hepatotoxicity depends on dose and time.<br>• The mechanisms involved include ER stress, apoptosis, disruption of glycolysis, and cell cycle arrest.<br>• In vivo, high doses (800 mg/kg) resulted in acute liver injury, elevated ALT levels, and apoptosis in hepatocytes.	[46]
HepG2 (hepatocellular carcinoma)	Human	TDCIPP, TPHP, TMPP, EHDPHP, TCEP, TBOEP, TNBP, TCIPP, TBBPA	0.1 µM to 25 µM	• OPFRs such as TMPP, TPHP, and TDCIPP led to significant lipid accumulation and mitochondrial dysfunction.<br>• Lipid metabolism genes (SREBP-1c and DGAT2) were upregulated, suggesting steatogenic effects.<br>• Aryl OPFRs exhibited stronger toxicity.	[37]

HepG2 (hepatocellular carcinoma)	Human	TPHP, TBP, TBOEP, TCPP	25 to 200 μM	• Cytotoxicity, DNA damage, and ROS levels increased in a dose-dependent manner.	[44]
HepG2 (hepatocellular carcinoma)	Human	TnBP, TPhP, TCPP, EHDPHP, TBOEP, TDCIPP, TEHP	0.23 to 2000 μM	• OPFRs demonstrated additive toxicity patterns with no evidence of synergism.<br>• TPhP exhibited the highest cytotoxicity, presenting an EC_{10} of 53.3 μM.<br>• Mixtures containing OPFRs showed cumulative toxicity effects, even at low doses.	[39]
LO2 (human liver cells)	Human	TCEP, TCPP, TPhP, TEHP, TMP, DnBP	1 μM to 100 μM	• TCPP and TPhP increased p53 expression in a dose-dependent manner, indicating DNA damage and the induction of apoptosis.<br>• TPhP demonstrated the strongest DNA binding affinity, disrupting the DNA structure.	[34]
Chicken embryonic cells	Chicken	TDCPP, TCPP	0.01 μM to 300 μM	• TDCPP exhibited cytotoxicity to hepatocytes (LC50: 60.3 μM) and neuronal cells (LC50: 28.7 μM) at concentrations ≥10 μM.<br>• Both OPFRs affected mRNA expression.<br>• Xenobiotic metabolism genes (CYP2H1, CYP3A37) were upregulated, while lipid metabolism genes were downregulated.	[51]
Mouse liver microsomes	Mouse	TDCPP, TPhP	0.1 μM to 1.0 μM	• TDCPP had higher bioaccumulation potential than TPhP.<br>• Key enzymes in TDCPP metabolism: CYP2E1, CYP2D6, CYP1A2, CYP2C19.<br>• CYP2E1 was the major enzyme in TPhP metabolism.	[53]

S2. Neurotoxicity

Cellular model	Organism	OPFRs used	Concentration	Main results	Ref
PC12 (rat pheochromocytoma cells)	Rat	TDCIPP, TCEP TCIPP	TDCIPP: 50 µM TCEP: 200 µM	• TDCIPP-induced apoptosis and alterations in neurodevelopmental gene expression were observed.<br>• TCEP produced cytotoxic effects and changes in gene expression.<br>• Slow elimination of some OPFRs in adipose tissue and brain was noted, suggesting potential bioaccumulation.	[50]
SH-SY5Y (human neuroblastoma cells)	Human	TDCPP, TPhP	2.5 µM to 20 µM	• No notable reduction in cell viability or mitochondrial dysfunction was observed at the tested concentrations.<br>• There was no release of inflammatory cytokines or significant effects on genes related to neural plasticity.	[54]
Primary microglia cells	Mouse	TDCPP	10–100 µM	• TDCPP exposure activates microglia, resulting in neuroinflammation.<br>• This process entails the release of cytokines (e.g., IL-1β, TNFα) and heightened expression of inflammatory markers (e.g., CD68, CD86).<br>• Neuroinflammation contributes to neuronal cell death and disrupts typical hippocampal development.<br>• Microglia are crucial as early responders, driving inflammation and adding to neurotoxicity.	[55]
Mouse embryonic stem cells	Mouse	TPHP, IPP, TMPP, TDCIPP	0.003 µM to 100 µM	• TPHP and TDCIPP reduced differentiation and exhibited cytotoxicity at higher concentrations.<br>• TMPP and IPP impacted differentiation but demonstrated limited toxicity at lower concentrations.	[29]

Rat cortical neurons (rat cortical neurons)<br>Human embryonic stem cell-derived neurons	Rat<br>Human	TPHP, IPP, TMPP, BPDP	0.003 µM to 100 µM	• Human neurons exhibited greater sensitivity than rat neurons.<br>• BPDP and TPHP inhibited neurite outgrowth in human neurons at lower concentrations than in rat neurons.	[29]
Rat neural networks (MEA) on microelectrode arrays	Rat	TMPP, BPDP, EHDPHP	0.003 µM to 100 µM	• TMPP, BPDP, and EHDPHP reduced neural network activity without causing significant cytotoxicity.<br>• Inflammation was absent, indicating functional effects rather than cytotoxic ones.	[29]
PC12 (rat pheochromocytoma cells)	Rat	TDCIPP	15 µM, 30 µM, and 60 µM	• Altered circRNA expression; 3,432 differentially expressed circRNAs; involvement of NF-κB signaling pathway and apoptosis.	[57]
PC12 (rat pheochromocytoma cells)	Rat	TCP, TPhP, EHDPHP, TDCP, TCPP, TCEP, TnBP, TPrP, TEP, TEHP, TBEP, TMP	25–100 µM	• OPFRs with aromatic or chlorinated alkyl groups inhibited OGT activity.<br>• TCP was the strongest inhibitor.<br>• OPFRs decreased protein O-GlcN acylation, increased ROS, calcium levels, cell proliferation, and autophagy.	[41]
3D rat brain organotypic model	Rat	IPP, TPHP, IDDP, TMPP	0.1–5 µM	• Stronger neurotoxicity than BDE-47.<br>• Reduced neurotransmitter levels (glutamate, GABA, NAA).<br>• Downregulated dopamine transporter.<br>• Induced inflammation and astrogliosis.<br>• Disrupted synaptic signaling and lipid metabolism.	[42]

S3. Endocrine disruption

Cellular model	Organism	OPFRs used	Concentration	Main results	Ref
CHO (Chinese Hamster Ovary cells) H295R cells (human adrenocortical carcinoma cells)	Hamster Human	BCIPP, BDCIPP, DPHP	0.1 to 10 µM	• BCIPP was the strongest ERα agonist.<br>• BDCIPP was the most effective MR antagonist.<br>• None of the metabolites exhibited cytotoxicity.<br>• Dose-dependent endocrine-disrupting effects were observed.	[28]
CHO-K1 (Chinese Hamster Ovary cells) COS-7 cells (monkey kidney fibroblast cells)	Hamster Monkey	TMP, TEP, TPrP, TBP, TCPP, TCEP, TBOEP, TDCPP, TEHP, TPhP, TCP	1.2 to 24 µM	• TPhP and TCP exhibited weak ERα/β agonistic activity.<br>• TDCPP, TPhP, and TCP demonstrated AR antagonism.<br>• Seven OPFRs functioned as PXR agonists.<br>• Cytotoxicity was detected only for TEHP and TCP at the highest concentrations.	[48]
H295R (human adrenocortical carcinoma cells) AREc32 (human osteosarcoma reporter cells)	Human	TCEP, TCIPP, TDCIPP, TPHP, DPHP, CDP, TIPPP, TMPP, EHDPHP, TEHP	12.5–100 µM	• TCEP decreased the expression of CYP19A1 and STAR, resulting in elevated testosterone levels.<br>• TPHP interfered with estradiol production and modified the expression of genes, including CYP19A1 and CYP11B2.<br>• TDCIPP stimulated an increase in testosterone production and upregulated steroidogenic genes.<br>• High hydrophobicity improved the interaction of OPFRs with cellular pathways.<br>• None of the OPFRs tested caused significant activation of the Nrf2 pathway, suggesting oxidative stress is not a primary mechanism of toxicity.	[33]

MCF7 (human breast adenocarcinoma cells)<br>MVLN (human breast cancer-derived reporter cells)	Human	14 OPFRs: TMP, TEP, TPHP, TiBP, THP, MPhP, DPK, MDPP, TPhP, CDP, TCP, IPPDP, IDPP, TnBP	1 to100 μM in the yeast assay.<br>0.5-1 μM in the E-SCREEN assay	• Most OPFRs promoted MCF7 proliferation in a concentration-dependent manner.<br>• The effects of alkyl-OPFRs correlated with hydrophobicity (LogKow), whereas aryl-OPFRs displayed no such trend.<br>• GPR30-mediated proliferation was observed for eight OPFRs.<br>• TPhP exhibited agonistic estrogenic activity.<br>• TPhP activated ERα-mediated transcription.<br>• Other OPFRs showed no detectable activity.	[32]

S4. Reproductive and developmental toxicity

Cellular model	Organism	OPFRs used	Concentration	Main results	Ref
GC-2 (mouse spermatocyte cells)	Mouse	TDCIPP	1, 3, 10, 30 µM	• Cell viability decreased in a dose- and time-dependent manner; ≥30 µM cut viability by >50%.<br>• Activation of the mitochondrial apoptosis pathway was confirmed through elevated caspase-3 and caspase-9 activity.<br>• TDCIPP disrupted mitochondria, causing fragmentation, reduced network integrity, altered membrane potential, and a 31.3% ATP decrease at 30 µM.<br>• TDCIPP caused ER stress with gene upregulation and structural damage shown by increased fluorescence.<br>• ROS levels rose, linking to mitochondrial dysfunction and apoptosis progression.<br>• TDCIPP triggered mitochondrial apoptosis by upregulating pro-apoptotic and downregulating anti-apoptotic proteins.	[58]
GC-2 (mouse spermatocyte cells)	Mouse	TPhP	6, 30, 60 µM	• Cell viability decreased in a dose- and time-dependent manner.<br>• TPhP, induced mitochondrial apoptosis via caspase-3/9 activation increased Bax and cytochrome c and decreased Bcl-2.<br>• ROS levels rose, resulting in mitochondrial dysfunction and cellular damage.<br>• Mitochondrial fragmentation, alterations in membrane potential, and reduced ATP levels were observed at concentrations of 30 µM and 60 µM.	[61]

				• DNA damage was evident at these concentrations, as indicated by increased pH2AX expression.<br>• Total antioxidant capacity (T-AOC) initially increased but was ultimately surpassed by excessive ROS at higher doses.	
MA-10 Leydig cells	Mouse	EDHP, IDDP, TPhP, BPDP, IPP, TMPP, TOCP	1–100 µM	• OPFRs caused greater mitochondrial activity reductions than BDE-47, with IPP being the most toxic and TPHP the least.<br>• OPFRs significantly reduced cell survival at lower concentrations compared to BDE-47.<br>• OPFRs increased oxidative stress, with higher superoxide production than BDE-47.<br>• OPFRs disrupted progesterone secretion, altering both basal and stimulated levels, unlike BDE-47 or TPHP.<br>• OPFRs altered key steroidogenesis-related gene expression, impacting hormone synthesis and signaling pathways.<br>• OPFRs showed higher overall toxicity than BDE-47, highlighting their stronger potential to disrupt Leydig cell function and reproductive health.	[30]
Human ovarian granulosa tumor cells	Human	TBOEP, TMPP, TCIPP, TDCIPP, TPHP, IDDPHP, TCEP, EHDPHP, TnBP, CDPP, BPDP	0.0009 µg/mL to 888 µg/mL	• Exposure caused structural and functional changes in the cells.<br>• Transcriptomic and lipidomic analyses showed disruptions in cholesterol synthesis and lipid metabolism.<br>• Findings suggest that real-world OPFR exposure can impact ovarian granulosa cells.	[31]

HTR-8/SVneo (immortalized human trophoblast cells)<br>JEG-3 (human choriocarcinoma cells)	Human	EHDPHP	10 μM, 20 μM, 30 μM	• In vitro, cell viability, migration, and angiogenesis were inhibited dose-dependently.<br>• Activation of LXRα reduced EHDPHP toxicity.	[35]
Mouse embryonic stem cells	Mouse	TPHP, IPP, TDCIPP, BPDP, TMPP, IDDP, TDCIPP, TCEP	0.003–100μM	• Cytotoxicity observed in 1–10 μM range.<br>• Decreased Goosecoid (GSC) expression disrupted differentiation.<br>• POD values: TPHP (41 μM), IPP (66 μM), TDCIPP (44 μM).	[29]
Human embryonic stem cells	Human	EHDPHP, IDDPP	0.0001-10μM	• EHDPHP and IDDPP significantly inhibited OCT4 expression (50% and 53% of control).<br>• Disrupted amniogenesis through ITGβ1 pathway, potentially linked to biochemical miscarriage.	[38]
Human embryonic stem cells	Human	TEP, TPHP, TCIPP	1 μM	• NEOs and OPFRs activated BMP4 signaling, affecting early differentiation and pluripotency maintenance, suggesting impact on early human development.	[59]

S5. Lung toxicity

Cellular model	Organisms	OPFRs used	Concentration	Main results	Ref
BEAS-2B (human bronchial epithelial cells)	Human	TCEP, TCPP, TDCPP, TBOEP,TPHP, EHDPHP, TEHP, TCP, TEP, TnBP, TiBP	3.10 to 544 ng/m³	• Compared to Guangzhou, OPFR concentrations were significantly higher in Taiyuan due to coal-driven emissions.<br>• Chloro-substituted OPFRs (TCEP, TCPP, TDCPP) dominated across both regions.<br>• Exposure to OPFRs resulted in significant cytotoxicity in BEAS-2B cells, with high concentrations decreasing cell viability by more than 50%.<br>• Oxidative stress assays revealed elevated ROS production, especially in Taiyuan samples, linking industrial and traffic emissions to toxicity.<br>• Estimated Daily Intake (EDI) values indicated low acute risk, but chronic exposure effects remain under-researched.	[36]
A549 (human lung adenocarcinoma cells)	Human	TCEP, TCPP	0.1 µM to 1000 µM	• TCPP exhibited significantly greater toxicity than TCEP, as indicated by lower IC50 values.<br>• Exposure to TCPP increased ROS levels in both intracellular and mitochondrial compartments, suggesting that oxidative stress is the primary mechanism of toxicity.<br>• Mitochondrial dysfunction was more pronounced in TCPP-treated cells.<br>• DNA damage induced by TCPP resulted in cell cycle arrest in the G1 phase and upregulation of the gene expressions for p53, p21, and Gadd45β.	[49]

				• TCPP initiated apoptosis, as evidenced by Bax expression and the sub-G1 apoptotic peak, while TCEP displayed milder effects.	
A549 (human lung adenocarcinoma cells)	Human	TPHP, TBP, TBOEP, TCPP	25 µM, 50 µM, 100 µM, and 200 µM	• OPFRs demonstrated concentration-dependent toxicity, becoming significant at 100 µM and increasing at 200 µM µM.<br>• TPHP and TBOEP exhibited the highest toxicity, decreasing cell viability by more than 30% at 200 µM.<br>• ROS production was highest with TBOEP and TCPP, displaying increases of 3.65-fold and 3.95-fold, respectively, at 200 µM.<br>• TPHP exposure led to the most significant DNA damage, causing a 1.79-fold rise in genotoxicity compared to controls.<br>• LDH leakage showed significant membrane damage, with TBOEP and TCPP causing the highest effects (31% and 22% increases in LDH activity) respectively.	[44]
A549 (human lung adenocarcinoma cells)	Human	TBOEP, TPhP, EHDPhP, TDCPP, TEHP, TCP, TCEP	25, 50, and 100 µM	• Dose-dependent toxicity; TPhP and TDCPP reduced cell viability at 100 µM.<br>• Disruptions in lipid metabolism (TG, DG, Cer, PI, PE-O) with increased TG accumulation.<br>• Oxidative stress, IL-8 upregulation, and apoptosis observed.<br>• Higher toxicity in aromatic (TPhP, EHDPhP, TCP) and chlorinated (TDCPP) OPFRs; milder effects in aliphatic (TEHP, TCEP) OPFRs.	Pyambri *et al.* (data pending publication)

S6. Other tissues

Cellular model	Organism	OPFRs used	Concentration	Main results	Ref
Caco-2 (human colon carcinoma cells)	Human	TPHP, TBP, TBOEP, TCPP	25, 50, 100, and 200 μM	• A significant reduction in cell viability occurred at 100 μM and 200 μM. • DNA damage was most severe in Caco-2 cells, with extensive strand breaks observed at higher concentrations. • LDH leakage indicated substantial membrane damage, particularly at 200 μM. • Caco-2 cells exhibited the highest sensitivity among the tested cell lines, underscoring their vulnerability to OPFR toxicity.	[44]
Caco-2 (human colon carcinoma cells)	Human	TDCPP, TCEP, TCPP	10 μM	• TDCPP altered gut microbiota, increasing infections and resistance. • TDCPP showed the strongest toxicity in Caco-2 cells. • Could potentially harm intestinal health.	[56]
Peripheral Blood Mononuclear Cells (PBMCs) (human immune cells)	Human	TCPP, TCEP	0.001 mM to 1 mM	• TCPP was more toxic than TCEP, showing significant viability reduction at 0.5 mM for TCPP and at 1 mM for TCEP. • TCPP caused marked morphological changes and necrosis compared to TCEP.	[48]
THP-1 (human monocytic leukemia cells)	Human	TPHP, TOCP	25 μM and 50 μM	• Impaired nucleic acid synthesis and energy metabolism and reduced ATP levels. • TPhP inhibited phagocytosis and adhesion. • TOCP activated mTOR signaling, enhancing immune responses. • Distinct immunomodulatory mechanisms observed.	[60]

HCECs<br>(human corneal epithelial cells)	Human	TPP	2 µM to 400 µM	• TPHP reduced cell viability and induced apoptosis.<br>• Dose-dependent reduction in mitochondrial membrane potential.<br>• Altered expression of apoptosis-related genes (Cyt c, Caspase-9, Caspase-3, Bcl-2, Bax).	[62]
HUVECs<br>(human vascular endothelial cells)	Human	TCPP	5 to 400 µM	• TCPP reduced cell survival and induced DNA damage.<br>• Increased ROS and mitochondrial dysfunction.<br>• Cell cycle alterations with more cells in apoptosis.	[52]

References

28. Zhang, Q.; Yu, C.; Fu, L.; Gu, S.; Wang, C. New Insights in the Endocrine Disrupting Effects of Three Primary Metabolites of Organophosphate Flame Retardants. Environ. Sci. Technol. 2020, 54, 4465–4474. https://doi.org/10.1021/acs.est.9b07874.

29. Behl, M.; Hsieh, J.H.; Shafer, T.J.; Mundy, W.R.; Rice, J.R.; Boyd, W.A.; Freedman, J.H.; Hunter, E.S.; Jarema, K.A.; Padilla, S.; et al. Use of alternative assays to identify and prioritize organophosphorus flame retardants for potential developmental and neurotoxicity. Neurotoxicol. Teratol. 2015, 52, 181–193. https://doi.org/10.1016/j.ntt.2015.09.003.

30. Schang, G.; Robaire, B.; Hales, B.F. Organophosphate Flame Retardants act as Endocrine-Disrupting Chemicals in MA-10 Mouse Tumor Leydig Cells Running title: Effects of OPFRs on MA-10 Leydig Cells Downloaded from, n.d. Available online: http://toxsci.oxfordjournals.org/ (accessed on).

31. Wang, X.; Rowan-Carroll, A.; Meier, M.J.; Yauk, C.L.; Wade, M.G.; Robaire, B.; Hales, B.F. House dust-derived mixtures of organophosphate esters alter the phenotype, function, transcriptome, and lipidome of KGN human ovarian granulosa cells. Toxicol. Sci. 2024, 200, 95–113. https://doi.org/10.1093/toxsci/kfae052.

32. Ji, X.; Li, N.; Ma, M.; Rao, K.; Wang, Z. In vitro estrogen-disrupting effects of organophosphate flame retardants. Sci. Total Environ. 2020, 727, 138484. https://doi.org/10.1016/j.scitotenv.2020.138484.

33. Rosenmai, A.K.; Winge, S.B.; Möller, M.; Lundqvist, J.; Wedebye, E.B.; Nikolov, N.G.; Johansson, H.K.L.; Vinggaard, A.M. Organophosphate ester flame retardants have antiandrogenic potential and affect other endocrine related endpoints in vitro and in silico. Chemosphere 2021, 263, 127703. https://doi.org/10.1016/j.chemosphere.2020.127703.

34. Li, F.; Cao, L.; Li, X.; Li, N.; Wang, Z.; Wu, H. Affinities of organophosphate flame retardants to tumor suppressor gene p53: An integrated in vitro and in silico study. Toxicol. Lett. 2015, 232, 533–541. https://doi.org/10.1016/j.toxlet.2014.12.006.

35. Zhang, Y.; Liang, J.; Gu, H.; Du, T.; Xu, P.; Yu, T.; He, Q.; Huang, Z.; Lei, S.; Li, J. Activation of LXRα attenuates 2-Ethylhexyl diphenyl phosphate (EHDPP) induced placental dysfunction. Ecotoxicol. Environ. Saf. 2023, 266, 115605. https://doi.org/10.1016/j.ecoenv.2023.115605.

36. Chen, Y.; Song, Y.; Chen, Y.J.; Zhang, Y.; Li, R.; Wang, Y.; Qi, Z.; Chen, Z.F.; Cai, Z. Contamination profiles and potential health risks of organophosphate flame retardants in PM2.5 from Guangzhou and Taiyuan, China. Environ. Int. 2020, 134, 105343. https://doi.org/10.1016/J.ENVINT.2019.105343.

37. Negi, C.K.; Bajard, L.; Kohoutek, J.; Blaha, L. An adverse outcome pathway based in vitro characterization of novel flame retardants-induced hepatic steatosis. Environ. Pollut. 2021, 289, 117855. https://doi.org/10.1016/j.envpol.2021.117855.

38. Xu, C.; Zhang, C.; Liu, Y.; Ma, H.; Wu, F.; Jia, Y.; Hu, J. Amniogenesis in Human Amniotic Sac Embryoids after Exposures to Organophosphate Flame Retardants. Environ. Health Perspect. 2023, 131, 047007. https://doi.org/10.1289/EHP11958.

39. Kim, S.; Kang, K.; Kim, H.; Seo, M. In Vitro Toxicity Screening of Fifty Complex Mixtures in HepG2 Cells. Toxics 2024, 12, 126. https://doi.org/10.3390/toxics12020126.

41. Gu, Y.; Yang, Y.; Wan, B.; Li, M.; Guo, L.H. Inhibition of O-linked N-acetylglucosamine transferase activity in PC12 cells—A molecular mechanism of organophosphate flame retardants developmental neurotoxicity. Biochem. Pharmacol. 2018, 152, 21–33. https://doi.org/10.1016/j.bcp.2018.03.017.

42. Hogberg, H.T.; de Cássia da Silveira E Sá, R.; Kleensang, A.; Bouhifd, M.; Ulker, O.C.; Smirnova, L.; Behl, M.; Maertens, A.; Zhao, L.; Hartung, T. Organophosphorus flame retardants are developmental neurotoxicants in a rat primary brainsphere in vitro model. Arch. Toxicol. 2021, 95, 207–228. https://doi.org/10.1007/s00204-020-02903-2.

43. Tastet, V.; Le Vée, M.; Kerhoas, M.; Zerdoug, A.; Jouan, E.; Bruyère, A.; Fardel, O. Interactions of organophosphate flame re-tardants with human drug transporters. Ecotoxicol. Environ. Saf. 2023, 263, 115348. https://doi.org/10.1016/j.ecoenv.2023.115348.

44. An, J.; Hu, J.; Shang, Y.; Zhong, Y.; Zhang, X.; Yu, Z. The cytotoxicity of organophosphate flame retardants on HepG2, A549 and Caco-2 cells. J. Environ. Sci. Health Part A 2016, 51, 980–988. https://doi.org/10.1080/10934529.2016.1191819.
46. Li, Z.; Tang, X.; Zhu, L.; Qi, X.; Cao, G.; Lu, G. Cytotoxic Screening and Transcriptomics Reveal Insights into the Molecular Mechanisms of Trihexyl Phosphate-Triggered Hepatotoxicity. Environ. Sci. Technol. 2020, 54, 11464–11475. https://doi.org/10.1021/acs.est.0c03824.
47. Kojima, H.; Takeuchi, S.; Itoh, T.; Iida, M.; Kobayashi, S.; Yoshida, T. In vitro endocrine disruption potential of organophos-phate flame retardants via human nuclear receptors. Toxicology 2013, 314, 76–83. https://doi.org/10.1016/j.tox.2013.09.004.
48. Mokra, K.; Bukowski, K.; Woźniak, K. Effects of tris(1-chloro-2-propyl)phosphate and tris(2-chloroethyl)phosphate on cell viability and morphological changes in peripheral blood mononuclear cells (in vitro study). Hum. Exp. Toxicol. 2018, 37, 1336–1345. https://doi.org/10.1177/0960327118783529.
49. Yuan, S.; Zhang, H.; Wang, S.; Jiang, X.; Ma, M.; Xu, Y.; Han, Y.; Wang, Z. Do the same chlorinated organophosphorus flame retardants that cause cytotoxicity and DNA damage share the same pathway? Ecotoxicol. Environ. Saf. 2024, 273, 116158. https://doi.org/10.1016/j.ecoenv.2024.116158.
50. Deepika, D.; Sharma, R.P.; Schuhmacher, M.; Kumar, V. Development of a rat physiologically based kinetic model (PBK) for three organophosphate flame retardants (TDCIPP, TCIPP, TCEP). Toxicol. Lett. 2023, 383, 128–140. https://doi.org/10.1016/j.toxlet.2023.06.006.
51. Crump, D.; Chiu, S.; Kennedy, S.W. Effects of tris(1,3-dichloro-2-propyl) phosphate and tris(1-chloropropyl) phosphate on cytotoxicity and mrna expression in primary cultures of avian hepatocytes and neuronal cells. Toxicol. Sci. 2012, 126, 140–148. https://doi.org/10.1093/toxsci/kfs015.
52. Saquib, Q.; Siddiqui, M.; Al-Khedhairy, A. Organophosphorus flame-retardant tris(1-chloro-2-propyl)phosphate is genotoxic and apoptotic inducer in human umbilical vein endothelial cells. J. Appl. Toxicol. 2021, 41, 861–873. https://doi.org/10.1002/jat.4158.
53. Chen, M.H.; Zhang, S.H.; Jia, S.M.; Wang, L.J.; Ma, W.L. In vitro biotransformation of tris(1,3-dichloro-2-propyl) phosphate and triphenyl phosphate by mouse liver microsomes: Kinetics and key CYP isoforms. Chemosphere 2022, 288, 132504. https://doi.org/10.1016/j.chemosphere.2021.132504.
54. Esplugas, R.; Linares, V.; Bellés, M.; Domingo, J.L.; Schuhmacher, M. In vitro neurotoxic potential of emerging flame retard-ants on neuroblastoma cells in an acute exposure scenario. Toxicol. Vitr. 2023, 87, 105523. https://doi.org/10.1016/j.tiv.2022.105523.
55. Zhong, X.; Wu, J.; Ke, W.; Yu, Y.; Ji, D.; Kang, J.; Qiu, J.; Wang, C.; Yu, P.; Wei, Y. Neonatal exposure to organophosphorus flame retardant TDCPP elicits neurotoxicity in mouse hippocampus via microglia-mediated inflammation in vivo and in vitro. Arch. Toxicol. 2020, 94, 541–552. https://doi.org/10.1007/s00204-019-02635-y.
56. Huang, W.; Jin, L.; Yin, H.; Tang, S.; Yu, Y.; Yang, Y. Assessments of the effects of tris(1,3-dichloro-2-propyl) phosphate (TDCPP) on human intestinal health from the aspects of intestinal flora changes and cytotoxicity to human cells. Sci. Total Environ. 2023, 894, 164823. https://doi.org/10.1016/J.SCITOTENV.2023.164823.
57. Li, Z.; Sun, J.; Liu, W.; Wu, J.; Peng, H.; Zhao, Y.; Qio, H.; Fang, Y. Changes in the circRNA expression profile of PC12 cells in-duced by TDCIPP exposure may regulate the downstream NF-κB pathway via the Traf2 gene. Chemosphere 2020, 254, 126834. https://doi.org/10.1016/j.chemosphere.2020.126834.
58. Feng, Y.; Wang, Z.; Duan, H.; Shao, B. Tris(1,3-dichloro-2-propyl) phosphate induces endoplasmic reticulum stress and mito-chondrial-dependent apoptosis in mouse spermatocyte GC-2 cells. Food Chem. Toxicol. 2024, 185, 114506. https://doi.org/10.1016/j.fct.2024.114506.
59. Zhang, S.; Yang, R.; Zhao, M.; Li, S.; Yin, N.; Zhang, A.; Faiola, F. Typical neonicotinoids and organophosphate esters, but not their metabolites, adversely impact early human development by activating BMP4 signaling. J. Hazard. Mater. 2024, 465, 133028. https://doi.org/10.1016/j.jhazmat.2023.133028.
60. Zhao, B.; Zheng, S.; Yang, G.; He, Z.; Deng, J.; Luo, L.; Li, X.; Luan, T. Rap1 and mTOR signaling pathways drive opposing immunotoxic effects of structurally similar aryl-OPFRs, TPHP and TOCP. Environ. Int. 2025, 195, 109215. https://doi.org/10.1016/J.ENVINT.2024.109215.
61. Feng, Y.; Shi, J.; Li, M.; Duan, H.; Shao, B. Evaluation of the cytotoxic activity of triphenyl phosphate on mouse spermatocytes cells. Toxicol. Vitr. 2023, 90, 109215. https://doi.org/10.1016/j.tiv.2023.105607.

62. Chen, C.; Cui, D.; Li, J.; Ren, C.; Yang, D.; Xiang, P.; Liu, J. Organophosphorus Flame Retardant TPP-Induced Human Corneal Epithelial Cell Apoptosis through Caspase-Dependent Mitochondrial Pathway. Int. J. Mol. Sci. 2024, 25, 4155. https://doi.org/10.3390/ijms25084155.
